# U-shape incision on prostate capsule: New intraperitoneal laparoscopic technique in simple prostatectomy: A case report

**DOI:** 10.1016/j.amsu.2021.102787

**Published:** 2021-09-04

**Authors:** Hamidreza Zia, Fatemeh Khatami, Seyed Mohammad Kazem Aghamir

**Affiliations:** Urology Research Center, Tehran University of Medical Sciences, Tehran, Iran

**Keywords:** Benign prostatic hyperplasia, Laparoscopy, Prostate, Prostatectomy, Transperitoneal, Simple prostatectomy

## Abstract

**Introduction:**

and importance: Laparoscopy is a known technique for simple prostatectomy using intraperitoneal or extraperitoneal approaches. In the present study, a novel method is suggested for easier access to the whole body and even the head of the adenoma.

**Case presentation:**

This method was performed on 6 patients with prostate hyperplasia. This was intraperitoneal method, started with 4–6 trocars and two transverse incisions on both sides of the endopelvic fascia. the prostate capsule is detected (using a Foley catheter balloon); the prostate capsule is transversely opened by a U-shape incision in a 0.5–1cm distance from the bladder neck to reach the adenoma and is dissected under the capsule to separate the capsule from the prostate. By ligashour capsule is opened laterally to the endopelvic fascia and separated from the prostate and gland is removed.

**Clinical discussion:**

The mean operation time was 114 minutes and the average intraoperative bleeding was 244.1 cc. IPSS (International Prostate Symptom Score), Q Max, and post-void residue, changes were significant with p-value = 0.003, respectively; however, pre and postoperative Hemoglobin was imprecise. The mean postoperative urinary leakage is reported at 22 cc.

**Conclusion:**

In short, this technique provides a better vision to prostate adenoma and the results can be compared with other laparoscopic approaches. Yet, larger sample sizes in different centers are required for determining realistic results.

## Highlights

• The new U-shape intraperitoneal simple laparoscopic prostatectomy method is quite uncomplicated and accessible for all surgeons.
• A novel method is suggested for the head of the adenoma.
• This technique can be compared with other laparoscopic approaches.

## 1 Introduction

**Fig. 1:**
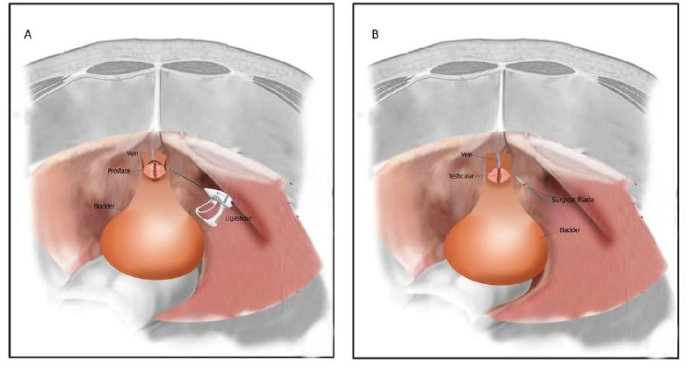
Prostatectomy by ligashour (A) and surgical blade (B).

Although several methods like transurethral prostate resection and laser thulium removal are suggested for treating unresponsive prostates or those causing kidney dysfunction, bladder stone or frequent infection and untreatable bleeding, open prostatectomy is still recommended in many centers around the world [1,2]. Laparoscopic technique could be currently used instead. Many studies have been done or are being done on the efficacy of this method [[3], [4], [5], [6], [7], [8]]. Both transperitoneal and extraperitoneal approaches of laparoscopic prostatectomy could be performed in laparoscopic open prostatectomy and both. (see Fig. 1)

## 2 Materials & methods

After Tehran university of medical sciences institutional research ethics committee approval *(IR.TUMS.SINAHOSPITAL.REC.1399.008), and the Iranian Registry of Clinical Trials (IRCT) code (20190624043991N15*) this study was performed in Sina hospital from 2018 to 2019. The study is adhered to SCARE guidelines [9]. Written informed consent was obtained from the patient for publication of this case report and accompanying images. A copy of the written consent is available for review by the Editor-in-Chief of this journal on request. This method was performed on 6 patients with prostate hyperplasia. An expert surgeon (urologist) runs the surgery and patients know that this current surgery method can be developed to decrease the surgery side effects and duration. All six patients have no history of drug history, family history including any relevant genetic information, and psychosocial history.

These selected patients had specific prostate weight and indications proper for open prostatectomy with no contraindications. IPSS (International Prostate Symptom Score) and Qmax were operated preoperatively and 3 months postoperatively. Also, patients' history, results of physical examination, routine laboratory tests (Creatinine, PSA (prostate-specific antigen)), and *Trans*-rectal Ultrasonography (TRUS) for evaluating prostate and its weight as well as postoperative complications such as general complications, cardiac problems, hemoglobin changes (preoperatively and 24 hours postoperatively) and patients’ hospital stay were examined. Following routine clinical examinations, preoperative TRUS was used for estimating prostate weight and there was an average of 82.66 ± 10.15 g (75–88g).

When it comes to technique, the patients undergo general anesthesia in the supine position with a head-and-body down tilt. NG-tube and 22F Foley catheter are placed for all patients. Legs are slightly opened (30°) and arms are fixed on both sides. Under direct vision, a 1–2cm incision is performed, the peritoneum is opened to insert a 10mm port inside, then, the abdomen is slowly (10–15 mmHg) filled with CO_2_ and the camera is placed through the umbilical port. Under direct vision, two 10mm ports are placed on a line between the umbilical cord and upper anterior spine lateral to the rectus muscle. Then, the peritoneum, bladder, and abdominal wall connection point are anteriorly opened as a transverse line entering the true pelvic cavity and the fourth trocar is placed in the suprapubic area for suction. In this method, the prostate capsule is detected (using a Foley catheter balloon); the prostate capsule is transversely opened by a U-shape incision in a 0.5–1cm distance from the bladder neck to reach the adenoma and is dissected under the capsule to separate the capsule from the prostate. By ligashour capsule is opened laterally to the endopelvic fascia and separated from the prostate like a tongue and the prostate is removed. After that, a three-way 22 F Foley catheter is inserted and the capsule is continuously sutured. Drain is placed next to the suture through the 5mm suprapubic port and the prostate tissue is put into the endo-sac and removed via the umbilical port. The drain clamp is opened and other ports are removed under direct vision and three port's fascia is sutured using nylon suture (Table 1). This method is recommended for surgeons willing to have a direct vision of the urinary tract connection and prostate upper border and those who care about the disconnection. On the first postoperative night, operation site wash was administered and all patients had catheters until the fifth day.

**Table 1:** Demographic and clinical information of 6 patients.

Variable	N	Minimum	Maximum	Mean	Std. Deviation
Age	6	57.00	82.00	68.66	9.54
Surgery long range (minutes)	6	90.00	140.00	114.33	17.95
Prostate Weight TRUS	6	60.00	85.00	70.66	10.15
Patient Weight	6	45.00	65.00	53.33	7.52
Creatinine	6	1.10	1.60	1.30	0.23
BUN	6	25.00	35.00	29.83	3.54
PSA	6	1.20	4.20	2.63	1.08
Prostate Wight	6	30.00	45.00	34.83	5.70
Maxim Flow rate before surgery	6	0.00	7.00	5.00	2.52
Maxim flow rate after surgery	6	22.00	26.00	23.83	1.47
IPSS Before Surgery	6	2.00	7.00	4.66	1.75
IPSS After Surgery	6	22.00	28.00	24.83	2.22
Urine Residue Before Surgery	6	35.00	102.00	65.36	27.32
Urine Residue After Surgery	6	10.00	50.00	20.00	15.16
Shelf Life	6	2.00	4.00	2.66	0.81
Bleeding (cc)	6	200.00	330.00	244.16	50.43
Hematuria	6	250.00	400.00	301.66	55.28
Hb before surgery	6	12.00	16.00	13.83	1.47
Hb after surgery	6	10.20	13.10	11.56	1.12
					

PSA: prostate specific antigen.

BUN: blood urea nitrogen.

TRUS: A transrectal ultrasound scan.

IPSS: The International Prostate Symptom Score.

## 3 Result

From February 2018 to September 2019, 6 simple laparoscopic prostatectomy operations were performed via the new U-shape incision in transperitoneal laparoscopy. The mean patients’ age was 68.66 ± 11.6 years (57–82 years.).

The mean duration of operation was 114.3 ± 17.95 minutes, (Range = 90–140 min); the average intraoperative bleeding was measured at 244.16 ± 54.16 cc (200–300 cc) and the mean weight of the tissue sample was 82.66 ± 10.15 g (75–88g). The average time of removing catheters was 3.1 ± 2.9 days. All patients were allowed to walk after hematuria resolution and none required transfusion (considering Hemoglobin changes). No postoperative cardiac complications, fever, or chills were observed. Urinary incontinence was not found in any patient after three months of follow-up. IPSS score was evaluated pre and postoperatively and the mean preoperative score of 24.83 ± 2.22 reduced to 4.66 ± 1.75 after the operation (p-value = 0.003).

The preoperative residual urine measured by catheter decreased from 65.36 ± 27.32 cc to 20.00 ± 15.16 cc (P VALUE = 0.29). The mean preoperative maximum flow rate was 5.00 ± 2.54/s increasing to 23.83 ± 1.47/s three months postoperatively (P VALUE = 31%). Other factors including pre and postoperative Hemoglobin changes showed no significant changes. The mean hospital stay was 3 days. The mean duration of operation was 114.33 ± 17.95 minutes with the mean intra and postoperative blood loss of 244 cc and 302 cc, respectively. Follow-up of one week, one month, and six months after surgery indicated the satisfying condition of patients.

## 4 Discussion

Medical and other surgical treatments of benign prostate hyperplasia such as transurethral resection of the prostate, transurethral incision of the prostate, open and laparoscopic prostatectomy are usually selected based on the specific conditions of patients. Prostate weight is a determining factor regarding the type of surgery. Even though several references are mentioning different weights for endoscopic and open prostatectomy, the appropriate one would be suggested by the surgeon considering his expertise and availability of the required equipment. However, in most well-known medical centers, open surgery is used for large and very large prostates instead of endoscopic procedures [[10], [11], [12]]. In our center, prostates≥70g by digital rectal examination are selected for open laparoscopic prostatectomy after ultra-sonographic confirmation.

Van Vel and his colleagues reported laparoscopic extraperitoneal Millin's prostatectomy for the first time on 18 patients. In their study average bleeding amount was 192 cc with a mean operation time of 145 minutes [4,13,14]. In our method, there were two extra incisions on the lateral sides at 1 h and 11 min, the dorsal vein was not blocked and retrigonazition was not performed; however, the operation time was less than Sotelo et al. approach reporting bleeding amount and operation time at 516 ml and 156 minutes, respectively and this can be due to not omitting the time for retrigonazition and blocking by ligashour lateral pedicures [15]. In some studies like Baumert et al. comparing simple laparoscopic and open prostatectomy, survival, duration, and presence of catheter as well as intraoperative bleeding were less in laparoscopic prostatectomy [11,16]. In 2006, Propriglia reported less postoperative bleeding in the laparoscopic prostatectomy group [[17], [18], [19]]. 1n 2019 Manfredi et al. run a study using extraperitoneal laparoscopic simple prostatectomy on 100 patients. In their study I-PSS, quality of life index, and maximum urine flow (Qmax) significantly improved when comparing preoperative and postoperative results. No significant differences were recorded in the I-PSS and I-PASS QoL index during 5-year follow-up [20,21].

As this study is methodically a case series, there are some limitations. This study tries to introduce this new technique and further randomized clinical trials are needed to compare this method with open prostatectomy in favor of outcomes and complications. In our patients and by using this new technique, IPSS, Q Max and residual urine volume reduction had better results, mentioning the fact that there is no restriction in using the technique for prostates with middle loops.

## 5 Conclusions

The new U-shape intraperitoneal simple laparoscopic prostatectomy method is quite uncomplicated and accessible for all surgeons; the surgeon can easily dissect the whole prostate, see the upper border and enter the incision in it. Nevertheless, this method requires a larger sample size to provide better evaluations.

## Ethics approval and consent to participate

All authors ensure our manuscript reporting adheres to CARE guidelines for reporting of case reports.

## Funding

There was no founding.

## Authors' contributions

All authors contribute equally.

## Consent to publish

Written informed consent was obtained from the patient for publication of this case report and accompanying images. A copy of the written consent is available for review by the Editor-in-Chief of this journal on request.

## Availability of data and material

All data will be provided on the request.

## Competing interests

All authors claim that there is no competing interest in this case report of surgery.

## Guarantor

Seyed Mohammad Kazem Aghamir.

## Provenance and peer review

Not commissioned, externally peer reviewed.

## Declaration of competing interest

Nor Applicable.
